# Factor XI localization in human deep venous thrombus and function of activated factor XI on venous thrombus formation and hemostasis

**DOI:** 10.1016/j.rpth.2025.102720

**Published:** 2025-03-03

**Authors:** Nobuyuki Oguri, Toshihiro Gi, Eriko Nakamura, Kazunari Maekawa, Eiji Furukoji, Hoshimi Okawa, Sho Kouyama, Saki Horiuchi, Akira Sawaguchi, Tatefumi Sakae, Minako Azuma, Yujiro Asada, Atsushi Yamashita

**Affiliations:** 1 Department of Pathology, Faculty of Medicine, University of Miyazaki, Miyazaki, Japan; 2 Department of Radiology, Faculty of Medicine, University of Miyazaki, Miyazaki, Japan; 3 Clinical Pharmacology, ONO Pharmaceutical Co., Ltd., Osaka, Japan; 4 Research Center of Speciality, ONO Pharmaceutical Co., Ltd., Osaka, Japan; 5 Division of Ultrastructural Cell Biology, Department of Anatomy, University of Miyazaki, Miyazaki, Japan; 6 Department of Diagnostic Pathology, Miyazaki Medical Association Hospital, Miyazaki, Japan

**Keywords:** factor X, factor XI, hemostasis, pathology, venous thromboembolism

## Abstract

**Background:**

Novel anticoagulants targeting coagulation factor (F)XI/activated FXI (FXIa) are currently under development. However, whether FXI is present in human deep vein thrombosis (DVT) and whether FXIa and activated FX (FXa) play different roles in venous thrombus formation and hemostasis remain unclear.

**Objectives:**

To determine the presence of FXI in DVT and the effects of direct oral FXIa and FXa inhibitors on venous thrombus formation and hemostasis in rabbits and on *in vitro* thrombus formation.

**Methods:**

We immunohistochemically assessed FXI localization in human-aspirated DVT (*n* = 15). Additionally, we compared thrombus formation induced by endothelial denudation and stenosis or stasis in the jugular vein and skin bleeding time and volume between rabbits treated with direct FXIa inhibitors (ONO-1600586) and FXa inhibitors (rivaroxaban). *Ex vivo* rabbit and human blood were perfused in a flow chamber under low-shear rates (70/s).

**Results:**

FXI was localized in all DVT, predominantly in fibrin-rich areas. The FXI immunopositive area in the nonorganizing area was greater than that in the organizing area. Although FXIa and FXa inhibitors comparably inhibited venous thrombus formation, FXIa inhibitors did not affect bleeding time or volume in rabbits. FXIa or FXa inhibitors mildly or strongly inhibited fibrin formation at low-shear rates, respectively. Furthermore, the FXIa inhibitor suppressed human FXIa activity, thrombin generation, and fibrin formation during perfusion.

**Conclusion:**

The pathologic findings of human DVT suggest FXI’s role in human DVT. FXIa inhibitors may inhibit less fibrin formation than FXa inhibitors and may explain the minor role of FXIa in hemostasis.

## Essentials

• The presence of FXI in venous thrombus and less bleeding in its inhibition is unclear.
• We assessed FXI localization in DVT and the function of FXIa in rabbits.
• FXI localized in human DVT, providing a rationale for FXI inhibition in human DVT.
• FXIa inhibitor inhibited less fibrin formation than factor Xa inhibitor under a low-shear rate.

## Graphical abstract

**Figure:**
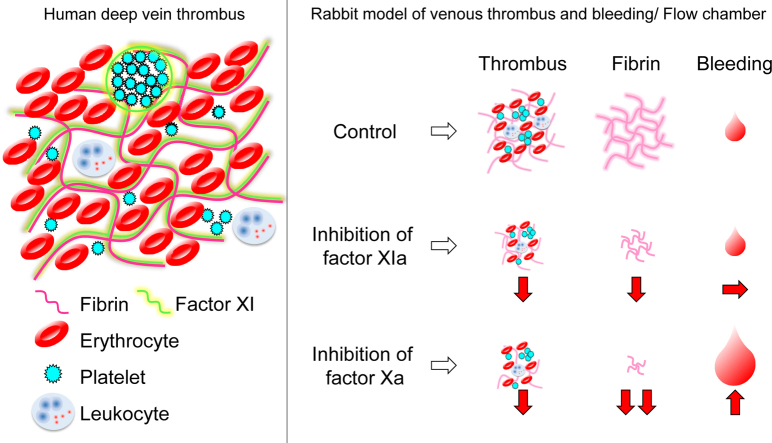

## 1 Introduction

Deep vein thrombosis (DVT) and pulmonary embolism, which are collectively termed venous thromboembolism (VTE), constitute major global health challenges. The estimates of VTE incidence range from 79 to 269 per 100,000 population [1]. Current oral anticoagulants that inhibit the protease thrombin or activated factor (F)X (FXa) or lower the plasma concentrations of their precursors, prothrombin or FX, can reduce the risks of VTE and ischemic stroke. However, their use has a certain risk of major or minor bleeding [2]. Even if the patients with atrial fibrillation receive low-dose direct oral anticoagulants, the incidence of major bleeding accounts for approximately 3% per year [3]. Therefore, developing anticoagulants without or with fewer bleeding complications is crucial.

FXI is a serine protease in the intrinsic pathway of coagulation, which is activated by thrombin, activated FXI (FXIa), and activated FXII (FXIIa) [4]. Elevated plasma FXI levels are associated with VTE and ischemic stroke [5,6], and FXIa is associated with an increased risk of recurrent VTE [7]. Furthermore, individuals with congenital FXI deficiency have a lower risk of cardiovascular and VTE events [8,9]. The age-adjusted incidence rate of VTE was two-thirds lower in individuals with FXI deficiency (FXIa activity ≤ 50%) than in those with normal FXIa activity [9]. In animal models, a genetic deficiency of FXI or inhibition of FXI and/or FXIa by antisense oligonucleotide, antibody, and small molecules reduced venous thrombus weight without increasing the bleeding time [[10], [11], [12], [13]]. However, ferric chloride [10,12] and silk thread [11] are not physiological initiators of venous thrombus formation, and a small-molecule FXIa inhibitor was intravenously (i.v.) administered [12]. Therefore, differences in venous thrombus formation and bleeding between oral administration of small-molecule FXa and FXIa inhibitors remain unelucidated. In addition, the exact mechanisms underlying the fewer bleeding phenotypes in FXI deficiency and FXIa inhibition are not clearly understood. Furthermore, the presence of FXI in human DVT has not yet been reported.

Therefore, this study aimed to investigate FXI localization in human DVT, the function of FXIa and FXa in venous thrombus formation and hemostasis in rabbits, and mural thrombus formation in a flow chamber system.

## 2 Methods

### 2.1 Aspirated thrombi from patients with DVT

The Ethics Committee of the University of Miyazaki approved the study protocol (approval number O-0684). Overall, 15 thrombi were obtained from 15 patients (8 men and 7 women; age range, 20-78 years; median, 54 years; Supplementary Table 1) with DVT who were diagnosed based on clinical symptoms, laboratory data, and clinical imaging findings. Thrombi were aspirated from the proximal portion using a guiding catheter (Guider Softip Guiding Catheter; Boston Scientific Japan). This catheter was placed from the popliteal vein to the iliac vein (10 cases) and the leg vein to the inferior vena cava (5 cases). X-ray venography revealed an extensive filling defect in the veins before thrombus aspiration and a reduced filling defect after thrombus aspiration. Finally, all aspirated thrombi were immediately fixed in 4% paraformaldehyde and embedded in paraffin for histologic evaluation.

### 2.2 Histologic findings of aspirated DVT

Briefly, 4-μm thick sections were stained with hematoxylin and eosin (HE) and morphologically assessed. Because the venous thrombi showed time-dependent changes, including fresh components, cell lytic changes, and organizing reactions, we defined the fresh component and cell lytic change as the nonorganizing area or the proliferation of endothelium-like flat cells and fibroblastic cells and fibrous matrix deposition as the organizing area [14,15]. Endothelialization was confirmed using immunohistochemistry for CD34 [16]. Finally, we compared the immunopositive areas described below between the fresh and organizing areas in each thrombus.

### 2.3 Immunofluorescence of venous thrombus

Immunofluorescence was performed to examine FXI localization in DVT. The DVT sections were stained with an anti-FXI antibody (sheep polyclonal, LS-B10243, LifeSpan BioSciences [LSBio], Inc), antifibrin antibody (mouse monoclonal, clone 59D8, EMD Millipore Corp), anti-CD42b antibody (rabbit monoclonal, Abcam PLC), antiglycophorin A antibody (mouse monoclonal, clone JC159, Dako/Agilent), anti-CD66b antibody (mouse monoclonal, clone 6/40c, BioLegend), anti-FXII antibody (mouse monoclonal, clone 2E2F11, Proteintech Group, Inc), or 4′,6-diamidino-2-phenylindole (DAPI; Nacalai Tesque, Inc), a DNA dye. CF488 conjugated donkey anti-sheep immunoglobulin (Ig) G (Biotium) and CF568 conjugated donkey anti-rabbit IgG (Biotium) or CF568 conjugated donkey anti-mouse IgG (Biotium) were used as secondary antibodies. Supplementary Figure 1 shows an immunopositive band for human FXI (HFXI 1111, Enzyme Research Laboratories Ltd), but not human prekallikrein (HPK 1302, Enzyme Research Laboratories Ltd), in Western blotting using the anti-FXI antibody (LS-B10243; LSBio). Microscopic images were captured using a photosensitive color charge coupled device (CCD) camera (DP-74, Olympus) under a 40× objective lens. We semiquantified the colocalized areas of FXI-fibrin, FXI-glycoprotein (GP) Ib, FXI-glycophorin A, FXI-CD66b, FXI-DAPI, and FXI-FXII using an image analyzing software (WinROOF, Mitani) [15]. The data are expressed as the ratio of colocalized areas to FXI immunopositive areas.

### 2.4 Immunohistochemistry for human DVT

Briefly, consecutive 4-μm slices of human-aspirated thrombi were immunohistochemically stained using antibodies against CD34 (mouse monoclonal, clone QBEnd10, Dako/Agilent), FXI (LSBio), fibrin (Millipore), antiplatelet GPIIb/IIIa (sheep monoclonal, Affinity Biologicals Inc), glycophorin A (Dako/Agilent), and CD66b (BioLegend). Next, the sections were stained with EnVision anti-mouse rabbit Ig (Dako/Agilent) or anti-sheep secondary antibodies (Jackson ImmunoResearch Inc). Horseradish peroxidase activity was visualized using 3, 3′-diaminobenzidine tetrahydrochloride, and the sections were mildly counterstained using Meyer’s hematoxylin. Immunostaining controls included nonimmune mouse IgG or nonimmune sheep serum rather than primary antibodies. Microscopic images were captured using a photosensitive color CCD camera (DS-Fi3, Nikon) under a 40× objective lens. The immunopositive areas of FXI, fibrin, glycophorin A, and GPIIb/IIIa in the nonorganizing and organizing areas were semiquantified using a color imaging morphometry system (WinROOF, Mitani). The detailed method has been described in a previous study [15]. Briefly, immunopositive areas were extracted as green areas using specific protocols based on the color parameters of hue, lightness, and saturation. The data were expressed as the mean ratio of positively stained areas per thrombus area in 3 fields [15]. Furthermore, the number of CD66b-immunopositive cells was counted in the 5 most densely stained fields under a 40× objective lens, and cell density was expressed as the number of immunopositive cells/mm^2^.

We also performed immunohistochemistry for FXI expression in human venous walls in patients with or without DVT in autopsy cases. The Ethics Committee of the University of Miyazaki approved the study protocol (approval number O-0681).

### 2.5 Immunoelectron microscopy by *in situ* nanogold labeling

We performed immunoelectron microscopy with *in situ* nanogold labeling to investigate the ultrastructure of FXI depositions. Micrographs of immunohistochemically stained sections for anti-FXI antibody (LSBio) with 3, 3′-diaminobenzidine tetrahydrochloride deposition were obtained. The coverslips of preselected microscope slides were removed, treated with 0.01% NaAuCl_4_.2H_2_O for 10 minutes, and incubated in a humid chamber at 37 °C for 12 hours or more. Sections labeled with nanogold particles were macerated with osmium and observed using scanning electron microscopy (Hitachi Tabletop Electron Microscope TM3000) [17].

### 2.6 Oral direct FXIa inhibitor and *in vitro* enzyme assay

We used the FXIa inhibitor ONO-1600586 (ONO Pharmaceutical Co, Ltd), which is a low-molecular-weight compound (a molecular weight of 507), to investigate the role of FXIa in venous thrombus formation and hemostasis.

The inhibitory activity of the compounds against FXIa, FXa, FXIIa, activated FIX (FIXa), activated FVII (FVIIa), plasma kallikrein, and thrombin was evaluated using the appropriate purified proteases and synthetic substrates. Furthermore, the hydrolysis rate of the chromogenic substrate by relevant proteases was continuously measured at 405 nm. Moreover, the inhibitory activity against each enzyme was calculated as percent inhibition using the equation described below:

%Inhibition=([ratewithoutcompound−ratewithcompound]/ratewithoutcompound)×100%.

Each half-maximal inhibitory concentration (IC_50_) value was determined by plotting the concentration of the compound against the percent inhibition.

Human FXIa (Haematologic Technologies Inc) activity was measured at an enzyme concentration of 0.1 U/mL in 150 mM NaCl (FUJIFILM Wako Pure Chemical Corp), 5 mM KCl (FUJIFILM Wako Pure Chemical Corp), 1 mg/mL polyethylene glycol (PEG; PEG6000, FUJIFILM Wako Pure Chemical Corp), and 50 mM 4-(2-hydroxyethyl)-1-piperazineethanesulfonic acid (Dojindo Laboratories)–NaOH (Nacalai Tesque Inc, pH 7.4) with 300 μM L-pyroglutamyl-L-prolyl-L-argininep-nitroaniline hydrochloride (Chromogenix S-2366, Diapharma).

Human plasma kallikrein (Enzyme Research Laboratories Ltd) activity was measured at an enzyme concentration of 0.605 mU/mL in 200 mM NaCl, 5 mg/mL PEG6000, and 100 mM phosphate (FUJIFILM Wako Pure Chemical Corp)–NaOH (pH 7.4) with 150 μM H-D-prolyl-L-phenylalanyl-L-arginine-p-nitroaniline dihydrochloride (Chromogenix S-2302, Diapharma).

Human FXa (American Diagnostica Inc) and thrombin (Sigma-Aldrich) activities were measured at enzyme concentrations of 0.18 U/mL and 0.12 U/mL, respectively, in the same buffer containing 150 mM NaCl, 2 mg/mL PEG6000, and 50 mM Tris (Nacalai Tesque Inc)-HCl (Nacalai Tesque Inc, pH 7.4), except that the reactions were started with 300 μM N-benzoyl-L-isoleucyl-L-glutamyl-glycyl-L-arginine-p-nitroaniline hydrochloride and its methyl ester (Chromogenix S-2222, Diapharma) and 300 μM Chromogenix S-2366 (Diapharma).

Human factor α-XIIa (Enzyme Research Laboratories Ltd) activity was measured at an enzyme concentration of 0.17 U/mL in 150 mM NaCl and 50 mM Tris-HCl (pH 7.4) with 300 μM Chromogenix S-2302 (Diapharma).

Human FIXa (American Diagnostica Inc) activity was measured at an enzyme concentration of 13 U/mL in 100 mM NaCl, 5 mM CaCl_2_ (Nacalai Tesque Inc), 30% ethylene glycol (Kanto Chemical Co, Inc), and 50 mM Tris-HCl (pH 7.4) with 3 mM Pefachrome FIXa 3960 (Leu-Ph′Gly-Arg-pNA, Pentapharm Ltd).

Human FVIIa activity was measured using recombinant human FVIIa (American Diagnostica Inc) in the presence of recombinant human tissue factor produced by ONO Pharmaceutical Co, Ltd, in a buffer containing 150 mM NaCl, 5 mM CaCl_2_, 0.5 mg/mL PEG6000, and 50 mM 4-(2-hydroxyethyl)-1-piperazineethanesulfonic acid–NaCl (pH 7.4) with 3 mM H-D-isoleucyl-L-prolyl-L-arginine-p-nitroaniline dihydrochloride (Chromogenix S-2288, Diapharma).

### 2.7 Rabbit model of jugular vein thrombosis by endothelial denudation and luminal stenosis and skin bleeding

The Animal Care Committee of the University of Miyazaki approved the animal study protocols (approval number 2019-532), which conformed to the Guide for the Care and Use of Laboratory Animals published by the U.S. National Institutes of Health. Overall, 71 healthy male Japanese white rabbits, purchased from Japan SLC Incorporation, weighing 2.8 to 3.3 kg, were fed a conventional diet.

The protocols of the venous thrombosis and skin bleeding models in rabbits were as follows (Supplementary Figure 2). First, fasting rabbits were orally administered a solvent as a control (*n* = 10), a FXIa inhibitor (ONO-1600586, 5 mg/kg, *n* = 10; 15 mg/kg, *n* = 10; and 50 mg/kg, *n* = 11), or a FXa inhibitor (rivaroxaban, 1.5 mg/kg, 5 mg/kg, and 15 mg/kg, *n* = 10 each) using a Nelaton catheter of 5 mm diameter (IZUMO Health Co, Ltd) 90 minutes before thrombus formation. The thrombus formation and bleeding were performed under general anesthesia through the subcutaneous injection of a mixture of medetomidine hydrochloride (0.08 mg/kg body weight), butorphanol tartrate (0.2 mg/kg), and midazolam (0.4 mg/kg) [18]. Additional anesthesia with one-third amount of the above-mentioned mixture of drugs was administered 80 minutes after the first injection, and all surgical operations proceeded under aseptic conditions.

Briefly, 90 minutes postadministration of the solvent, ONO-1600586, or rivaroxaban, venous thrombi were induced in rabbit jugular veins through endothelial denudation using a 3F balloon catheter (Edwards Lifesciences), according to a previous study [18], and the method was modified to induce luminal stenosis. A 2 mm polyethylene tube was placed outside the vessel. Both the jugular vein and polyethylene tube were ligated, and the tube was removed (Supplementary Figure 3) [19]. Next, 3 hours after thrombus formation and stenosis, the rabbits were infused with heparin (500 U/kg, i.v.) and then euthanized with an overdose of pentobarbital (60 mg/kg, i.v.). Subsequently, the animals were perfused with 50 mL phosphate-buffered saline (0.01 mol/L), and the jugular veins were sampled. The venous thrombi were immediately removed from the venous wall and weighed. Finally, the thrombus was fixed in 4% paraformaldehyde for 24 hours and embedded in paraffin for histologic evaluation.

Ninety minutes after administering the solvent, rivaroxaban (1.5, 5, and 15 mg/kg), and ONO-1600586 (5, 15, and 50 mg/kg), skin bleeding was induced using 2 lines of 3 cm length incisions on both legs of the rabbit. Next, wet Kimwipes (Kimberly-Clark Corp) were placed on the injured lesions, and the bleeding time was measured. To measure the bleeding volume, we collected the Kimwipes into a falcon tube filled with 20 mL of saline each, 1 to 2 minutes for 30 minutes, and then removed the Kimwipes. The collected blood was centrifuged at 900 × *g* for 5 minutes at room temperature, and the supernatant was discarded. One milliliter of distilled water was added to the pellet and then hemolyzed. The bleeding volume was estimated using a spectrophotometer at 540 nm (SpectraMax 190 Microplate Reader, Molecular Devices, LLC). A standard curve was generated through the sequential dilution of rabbit blood. Finally, the number of erythrocytes was measured using a hematology analyzer (XP-300, Sysmex).

### 2.8 Coagulation parameters

Blood samples were collected from the central ear arteries of rabbits before and 75 minutes and 4.5 hours after the administration of the solvent, ONO-1600586, and rivaroxaban in 3.8% sodium citrate (9:1 v/v) using 21-gauge needles. Plasma samples were centrifuged at 1500 × *g* for 15 minutes at room temperature. Prothrombin time (PT) and activated partial thromboplastin time (aPTT) were measured using a coagulation timer (KC-1 Delta, Tcoag Ireland Ltd) and reagents (aPTT: ACTIN, Siemens; PT: THROMBOCHECK PT+, Sysmex).

### 2.9 Stasis-induced thrombus formation in rabbit jugular veins

Because DVT is considered to be formed mostly by stasis, we examined the roles of FXIa and FXa inhibitors in stasis-induced thrombus formation in the rabbit jugular vein. The Animal Care Committee of the University of Miyazaki approved the animal study protocols (approval number 2019-532-5), which conformed to the Guide for the Care and Use of Laboratory Animals published by the U.S. National Institutes of Health. Overall, 24 healthy male Japanese white rabbits, purchased from Japan SLC Incorporation, weighing 2.8 to 3.1 kg, were fed a conventional diet.

The protocols of venous thrombosis by stasis were as follows (Supplementary Figure 4). First, fasting rabbits were orally administered a solvent as a control (*n* = 8), a FXIa inhibitor (ONO-1600586, 50 mg/kg, *n* = 8), or a FXa inhibitor (rivaroxaban, 15 mg/kg, *n* = 8) using a Nelaton catheter of 5 mm diameter (IZUMO Health Co, Ltd) 60 minutes before thrombus formation. The process of thrombus formation was performed under general anesthesia through the subcutaneous injection of a mixture of medetomidine hydrochloride (0.08 mg/kg body weight), butorphanol tartrate (0.2 mg/kg), and midazolam (0.4 mg/kg) [18].

Briefly, 60 minutes postadministration of the solvent, ONO-1600586, or rivaroxaban, venous thrombi were induced in rabbit jugular veins by ligations and luminal stenosis. To induce luminal stenosis, a 1 mm polyethylene tube was placed outside the vessel. Both the jugular vein and polyethylene tube were ligated 4 cm upstream of the ligature, and the tube was removed (Supplementary Figure 5). Next, 6 hours after stasis in the jugular vein, the rabbits were infused with heparin (500 U/kg, i.v.). They were then euthanized with an overdose of pentobarbital (60 mg/kg, i.v.), and the jugular veins were sampled. In this model, the animals were not perfused with phosphate-buffered saline because the thrombi were fragile. The venous thrombi were immediately removed from the venous wall and weighed. Finally, the thrombus was fixed in 4% paraformaldehyde for 24 hours and embedded in paraffin for histologic evaluation.

Blood samples were collected from the central ear arteries of the rabbits before and 1, 3, and 7 hours after administering the solvent, ONO-1600586, and rivaroxaban in 3.8% sodium citrate (9:1 v/v) using 21-gauge needles. Plasma samples were centrifuged at 1500 × *g* for 15 minutes at room temperature. PT and aPTT were measured using a coagulation timer (KC-1 Delta, Tcoag Ireland Ltd) and reagents (aPTT: ACTIN, Siemens; PT: THROMBOCHECK PT+, Sysmex). Data are expressed as a ratio of PT and aPTT to those before administration.

### 2.10 Histology and immunohistochemistry of jugular vein thrombus

Paraffin-embedded sections (4 μm thick) of the rabbit jugular vein thrombus were stained with HE and morphologically assessed. Consecutive sections of thrombi were immunohistochemically stained with antibodies against fibrin (Millipore) and GPIIb/IIIa (Affinity Biologicals Inc). Images were digitized using a photosensitive color CCD camera (BX51, OLYMPUS). Finally, the area of the red-stained erythrocytes in HE staining and the immunopositive areas for fibrin and GPIIb/IIIa were semiquantified using a color imaging morphometry system (WinROOF, Mitani).

### 2.11 *Ex vivo* rabbit blood perfusion with flow chamber system and visualization of the thrombi on the collagen surface

Blood from rabbits 75 minutes postadministration of a solvent as a control (*n* = 5), a FXIa inhibitor (ONO-1600586, 50 mg/kg, *n* = 5), or a FXa inhibitor (rivaroxaban, 15 mg/kg, *n* = 5) was collected using 21-gauge needles into plastic syringes containing corn trypsin inhibitor (Molecular Innovations; final concentration 20 μg/mL) and a specific FXa inhibitor, Arixtra (Organon Sanofi-Synthelabo, final concentration 6 μg/mL). Anticoagulated whole blood was stored at room temperature and used for perfusion studies within 1 hour of collection. Next, mepacrine (quinacrine dihydrochloride, Sigma-Aldrich, final concentration 5 μM) was added to the blood before perfusion for platelet labeling, which enables the visualization of platelet-surface interaction through epifluorescence videomicroscopy [13]. Collagen from the horse tendon (Collagen Reagent HORM, Takeda Pharmaceutical, 20 μg/mL) was immobilized on glass coverslips (24 × 50 mm) in parallel-plate flow chambers, as previously described [13]. Blood samples were perfused through the chamber using a syringe pump (KDS100, KD Scientific Inc) at a constant flow rate (15 mL/h) to achieve wall shear rates of 70/s. Platelets interacting with the immobilized collagen were visualized using an inverted stage epifluorescence microscope (IX71, OLYMPUS). Images were captured at 3, 6, and 9 minutes after perfusion with a photosensitive color CCD camera (DP70, Olympus), and surface covering areas were semiquantified using a morphometric analysis system (WinROOF, Mitani).

After perfusion, coverslips were immediately fixed in 95% alcohol for 1 hour at room temperature, stained using antibodies for fibrin (Millipore), and faintly counterstained with Meyer’s hematoxylin. The microscopic images were digitized using a photosensitive color CCD camera (DS-Fi3, Nikon), and the immunopositive areas of the images were semiquantified for each antibody using a color imaging morphometry system (WinROOF, Mitani). Finally, the number of attached cells with multilobulated nuclei was counted as the neutrophil number in 5 fields under a 20× objective lens and expressed as the number of neutrophils per mm^2^.

### 2.12 *In vitro* human blood perfusion and measurement of blood coagulation before and after the perfusion

The Ethics Committee of the University of Miyazaki approved this study (approval number O-1160). The Collagen Reagent HORM (Takeda Pharmaceutical, 20 μg/mL) and recombinant tissue factor (Dade Innovin, ×1000, Siemens) were immobilized on glass coverslips (24 × 50 mm) in parallel-plate flow chambers, as described above. Blood from healthy human volunteers who were not on medication was collected using 21-gauge needles into plastic syringes containing a corn trypsin inhibitor (Molecular Innovations, final concentration 10 μg/mL). Anticoagulated whole blood was used for the perfusion. The solvent (*n* = 10), FXIa inhibitor (ONO-1600586, 3 μmol/L, *n* = 10) or FXa inhibitor (rivaroxaban, 1 μmol/L, *n* = 10), and mepacrine (Sigma-Aldrich, final concentration 5 μM) were added to the blood before perfusion. Blood samples were perfused through the chamber using a syringe pump (KDS100, KD Scientific Inc) at a constant flow rate (15 mL/h) to achieve wall shear rates of 70/s. Finally, the surface covering area, fibrin-immunopositive area, and the number of neutrophils were analyzed as described above.

Immunofluorescence was performed to examine FXI localization in *in vitro* thrombi. The coverslips were stained with an anti-FXI antibody (LSBio), antifibrin antibody (Millipore), anti-CD42b antibody (Abcam), or anti-CD66b antibody (BioLegend). CF488 conjugated donkey anti-sheep IgG (Biotium) and CF568 conjugated donkey anti-rabbit IgG (Biotium) or CF568 conjugated donkey anti-mouse IgG (Biotium) were used as secondary antibodies. The immunofluorescent images were visualized using a spinning disk confocal microscope (SpinSR10, Evident Scientific).

The pre-and postperfused blood samples were collected into a tube containing 3.8% sodium citrate anticoagulant (9:1 v/v). Plasma samples were centrifuged at 1500 × *g* for 15 minutes at room temperature. FXIa and FXa activities were measured using an automatic blood coagulation analyzer (ACL-TOP, Werfen), as well as FXI-deficient plasma (HemosIL Factor XI Deficient Plasma and HemosIL SynthASil APTT, Werfen) and FX-deficient plasma (HemosIL Factor X Deficient Plasma and HemosIL RecombiPlasTin, Werfen). The plasma levels of prothrombin fragment 1 + 2 were measured using an enzyme-linked immunosorbent assay (Enzygnost F1 + 2 Monoclonal, Siemens).

### 2.13 Statistical analysis

Data analysis was performed using GraphPad Prism 9 (GraphPad Software Inc). All procedures were performed using a blinded experimental design. All data are expressed as means ± SD or median and IQR when the variance was skewed. Differences between or among individual groups were statistically evaluated using the Mann–Whitney U-test, Kruskal–Wallis test with Dunn’s multiple comparison test, or a 1- or 2-way analysis of variance (anova) with Tukey’s multiple comparison test as appropriate. Statistical significance was considered at *P* ˂ .05 (*n* indicates the number of samples). We have indicated the statistics in the legends of each figure.

## 3 Results

### 3.1 Localization of FXI in human DVT

**Figure 1:**
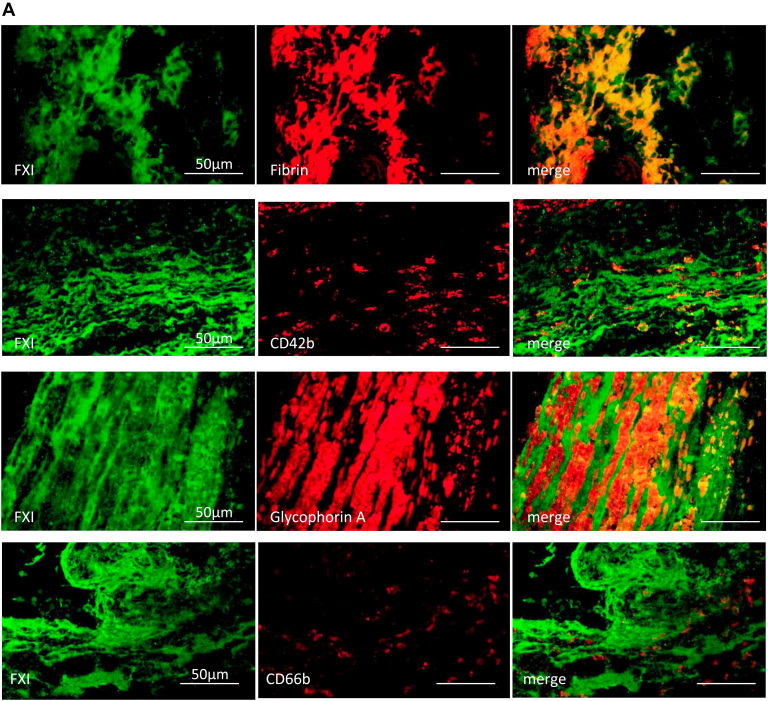

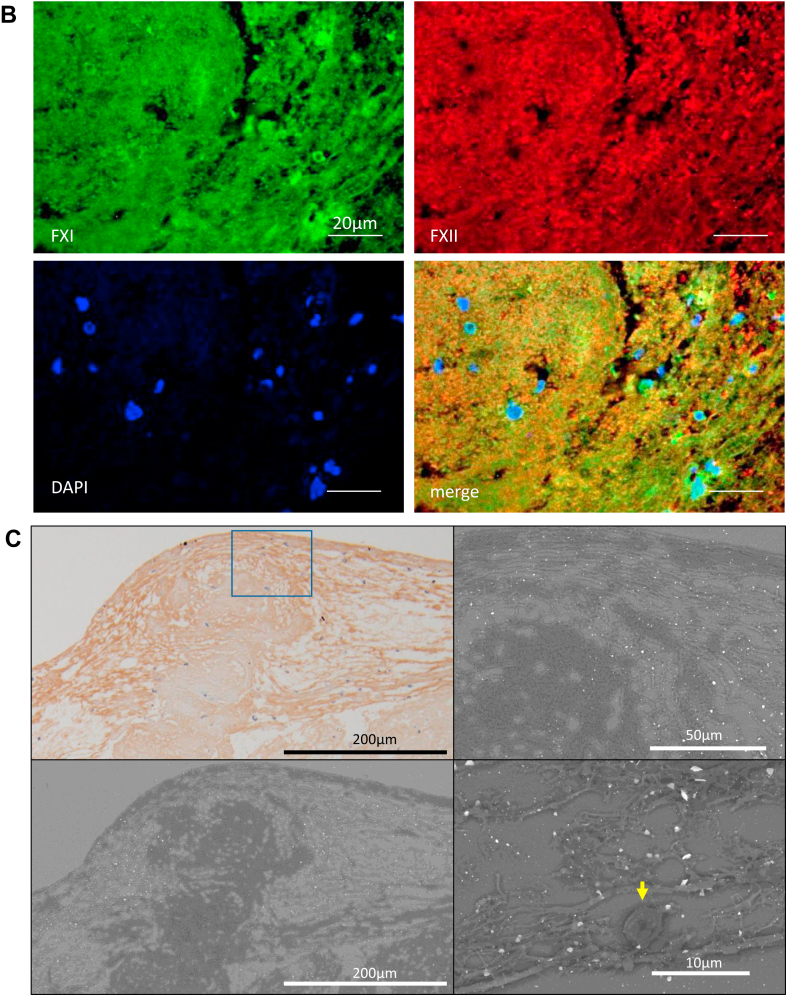

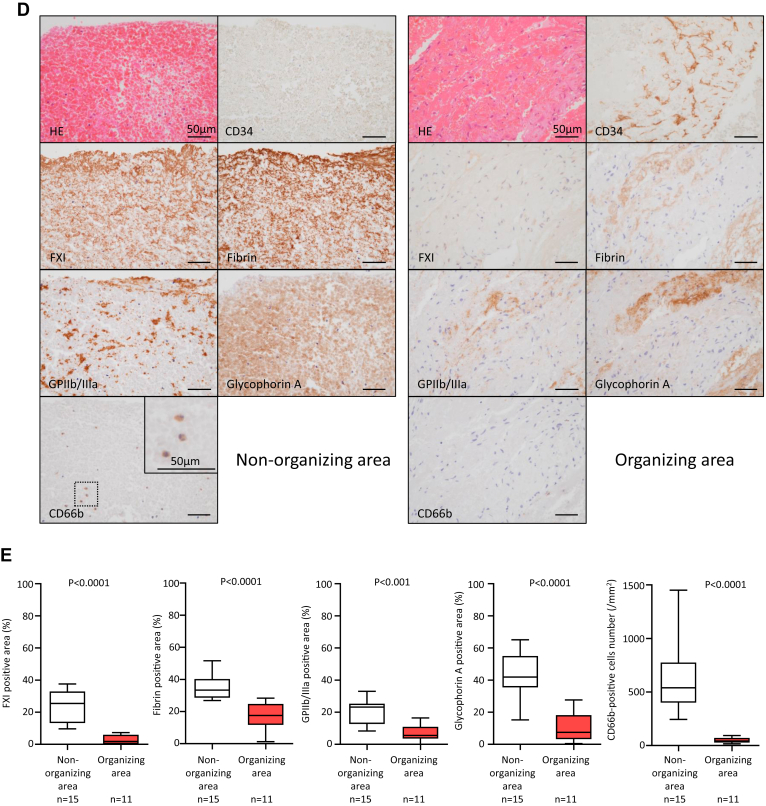
Presence of factor (F)XI in human deep vein thrombosis (DVT). (A) Representative immunofluorescent images of fresh components of DVT. The upper row shows CF488-labeled FXI (green), CF568-labeled fibrin (red), and merged images. The second row shows CF488-labeled FXI, CF568-labeled glycophorin A, and merged images. The third row shows CF488-labeled FXI, CF568-labeled CD42b, and merged images. The lower row shows CF488-labeled FXI, CF568-labeled CD66b, and merged images. FXI was closely localized with fibrin. Glycophorin A, a marker of erythrocytes, was predominantly surrounded by FXI. FXI was partly localized with CD42b, a marker of platelets, but hardly localized with CD66b, a marker of neutrophils. (B) Representative immunofluorescent images of fresh components of DVT show CF488-labeled FXI, CF568-labeled FXII, 4,6-diamidino-2-phenylindole (DAPI, a DNA marker), and merged images. FXI was partly localized with DNA and FXII. (C) Representative images of *in situ* nanogold labeling and electron microscopy of fresh components of DVT. The left upper image shows an immunohistochemical image of FXI. Nanogolds accumulate at the 3, 3′-diaminobenzidine tetrahydrochloride deposition site against anti-FXI antibody (left lower). Nanogold deposition was localized on a fibrin-like mesh network but not on the neutrophils (arrow). (D) Representative immunohistochemical images in fresh and organizing areas of DVT. The organizing areas were confirmed by the presence of CD34-immunopositive cells, corresponding to endothelialization/organization, while the fresh area was confirmed by the absence of CD34-immunopositive cells. FXI localized fibrin- and erythrocyte-rich fresh areas. In the fresh area, aggregated clusters of platelets and sparsely distributed neutrophils are present. The inset in CD66b represents a high-magnification image of the dashed square. In the organizing area, immunoreaction for FXI, fibrin, glycoprotein (GP) IIb/IIIa, glycophorin A, or CD66b is modest or focal. (E) Immunopositive area for FXI, fibrin, GPIIb/IIIa, and glycophorin A, and CD66b-immunopositive cell density in nonorganizing (CD34-immunonegative) and organizing (CD34-immunopositive) areas (Mann–Whitney U-test). HE, hematoxylin and eosin.

All 15 thrombi were immunopositive for FXI. We performed double immunofluorescence for FXI and fibrin, platelet CD42b, erythrocyte glycophorin A, neutrophil CD66b, DNA dye DAPI, or FXII. Immunofluorescent images showed that FXI was closely localized to fibrin rather than CD42b, glycophorin A, or CD66b (Figure 1A). In addition, FXI focally colocalized with DAPI and FXII (Figure 1B). The median and range of the immunofluorescent colocalized area of FXI-fibrin, FXI-FXII, FXI-glycophorin A, FXI-CD42b, FXI-DAPI, and FXI-CD66b were 67% (52%-77%), 26% (20%-48%), 14% (12%-20%), 2.2% (2.1%-7.6%), 1.2% (0.82%-1.6%), and 0.08% (0.018%-0.26%), respectively. *In situ* nanogold labeling visualizes the immunoenzymatic products on paraffin sections under a scanning electron microscope [17]. With *in situ* nanogold labeling for the FXI immunohistochemical paraffin section, FXI nanogold deposition was localized on a fibrin-like mesh network but not on the neutrophils that retained their shape (Figure 1C). We measured the immunopositive areas of FXI, fibrin, platelets, erythrocytes, and neutrophils to examine the extent of thrombus components and FXI in the nonorganizing and organizing areas. The organizing area was confirmed by the presence of CD34-immunopositive cells, which corresponds to endothelialization. All 15 thrombi were immunopositive for glycophorin A (an erythrocyte marker), GPIIb/IIIa (a platelet marker), fibrin, and CD66b (a neutrophil marker; Figure 1D). The areas immunopositive for FXI, fibrin, platelets, erythrocytes, and CD66b in the nonorganizing area were larger than those in the organizing area (Figure 1E).

We also analyzed FXI expression in the venous wall of autopsy cases with or without DVT. DVT was immunopositive for FXI; however, no immunoreaction for FXI was observed in the venous wall (Supplementary Figure 6).

### 3.2 Characterization of a FXIa inhibitor (ONO-1600586)

We used a FXIa inhibitor, ONO-1600586, to evaluate the function of FXIa. ONO-1600586 prolonged aPTT but did not affect PT in both humans and rabbits. Additionally, ONO-1600586 concentration at aPTT doubling time was 0.60 μM and 0.61 μM for humans and rabbits, respectively. ONO-1600586 did not double PT at plasma concentrations of 33 μM in humans and rabbits. ONO-1600586 inhibited human FXIa (IC_50_, 0.0020 μM) and weakly human kallikrein (IC_50_, 0.12 μM) but did not inhibit human thrombin, FVIIa, FIXa, FXa, FXIIa, plasmin, urokinase, or tissue plasminogen activator (IC_50_, >25 μM; Supplementary Table 2).

### 3.3 Oral administration of FXIa inhibitor prolongs aPTT rather than PT in rabbits, whereas oral administration of FXa inhibitor prolongs both PT and aPTT

**Figure 2:**
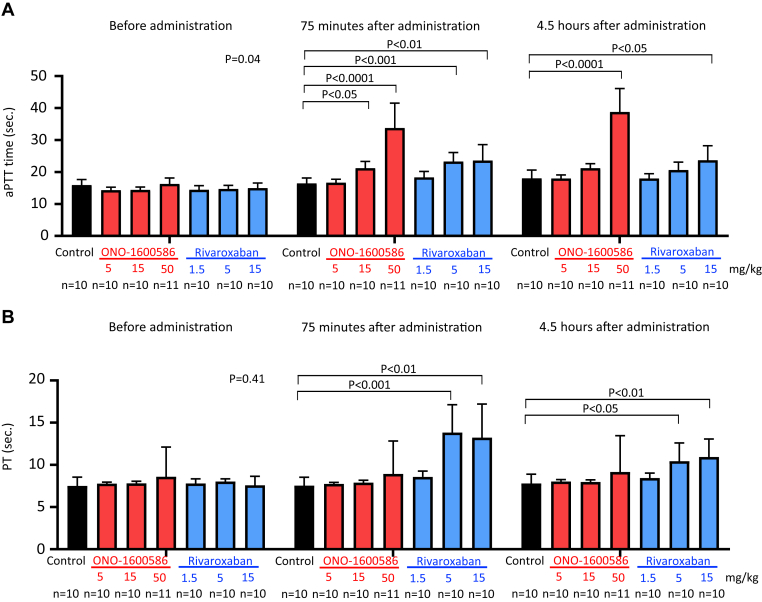
Activated partial thromboplastin time (aPTT) and plasma prothrombin time (PT) before and after oral administration of ONO-1600586 (activated factor [F]XI inhibitor) and rivaroxaban (activated FX inhibitor). (A) aPTT before and 75 minutes (before thrombus formation and bleeding test) and 4.5 hours (3 hours after thrombus formation) after oral administration of the solvent (control), ONO-1600586, and rivaroxaban (Kruskal–Wallis test with Dunn’s multiple comparisons test). aPTT before administration showed significant differences among the groups. However, there was no significant difference between the control and others (FXIa and FXa inhibitors) or between each group and every other group in Dunn’s multiple comparison test; *n* means the number of animals. (B) PT before and 75 minutes (before thrombus formation and bleeding test) and 4.5 hours (3 hours after thrombus formation) after oral administration of the control, ONO-1600586, and rivaroxaban (Kruskal–Wallis test with Dunn’s multiple comparisons test; *n* means the number of animals).

We evaluated PT and aPTT before and after the oral administration of the solvent, FXIa inhibitor (ONO-1600586), and FXa inhibitor (rivaroxaban) during venous thrombus formation and measured the bleeding time. aPTT, but not PT, at baseline, showed significant differences among the groups. However, there was no significant difference between the control and others (FXIa and FXa inhibitors) or between each group and every other group in Dunn’s multiple comparison test (Figure 2A). ONO-1600586 dose-dependently prolonged aPTT, but not PT, to 75 minutes. ONO-1600586 (50 mg/mL) prolonged aPTT, but not PT, for 4.5 hours. Rivaroxaban dose-dependently prolonged both PT and aPTT to 75 minutes. Rivaroxaban (15 mg/mL) prolonged both PT and aPTT for 4.5 hours. However, oral administration of the solvent did not affect the PT or aPTT at either time point (Figure 2A, B).

### 3.4 Oral administration of FXIa and FXa inhibitors suppresses venous thrombus formation by endothelial denudation and luminal stenosis in rabbits

**Figure 3:**
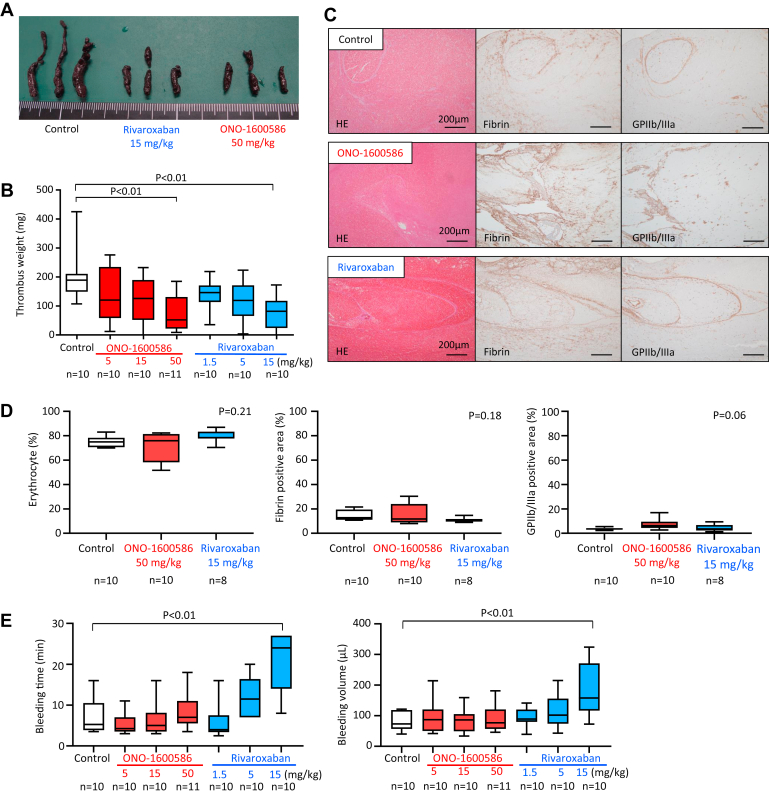
Different contributions of activated factor (F)XI and activated FX to venous thrombus formation with endothelial denudation and luminal stenosis and skin bleeding in rabbits. (A) Representative macroscopic images of the postfixed venous thrombi. Thrombi are dark reddish in color in each group. Thrombi of rivaroxaban and ONO-1600586 administration groups are smaller than those of the control (solvent) group. (B) Weight of unfixed venous thrombus in control, ONO-1600586, or rivaroxaban administration groups. Oral administration of ONO-1600586 (50 mg/kg) and rivaroxaban (15 mg/kg) similarly reduced venous thrombus weight compared with that of the control. Each group was compared with the control group (Kruskal–Wallis test with Dunn’s multiple comparison test; *n* means the number of animals). (C) Representative histologic and immunohistochemical images of venous thrombus in control, ONO-1600586, or rivaroxaban groups. All thrombi are rich in erythrocytes and contain fibrin and platelets (glycoprotein [GP] IIb/IIIa). (D) The areas of erythrocytes or immunopositive areas for fibrin or GPIIb/IIIa in venous thrombus (Kruskal–Wallis test; *n* means the number of histologic sections). (E) Bleeding time and bleeding volume after oral administration of the control, ONO-1600586, and rivaroxaban. Rivaroxaban dose-dependently prolonged bleeding time (left) and increased bleeding volume (right). Oral administration of ONO-1600586 did not affect bleeding time or volume. Each group was compared with the control group (Kruskal–Wallis test with Dunn’s multiple comparisons test; *n* means the number of histologic sections). HE, hematoxylin and eosin.

Venous thrombus formation was induced by endothelial denudation and luminal stenosis in the rabbit jugular vein after oral administration of the solvent, FXIa inhibitor (ONO-1600586), and FXa inhibitor (rivaroxaban). Figure 3A shows the representative macroscopic images of rabbit jugular vein thrombi in the control, FXIa inhibitor, and FXa inhibitor groups. The postfixed thrombi were dark reddish. Oral administration of ONO-1600586 (50 mg/kg) and rivaroxaban (15 mg/kg) significantly reduced the venous thrombus weight compared with that of the control (Figure 3A, B). Doses of 5 and 15 mg/kg ONO-1600586 and 1 and 5 mg/kg rivaroxaban tended to reduce the venous thrombus weight; however, this was not statistically significant.

Next, we histologically examined whether FXIa and FXa inhibition affected thrombus composition. All venous thrombi comprised erythrocytes, fibrin, and platelets (Figure 3C). Although the immunopositive area of fibrin tended to be smaller in the rivaroxaban group than in the control and ONO-1600586 groups, the areas of erythrocytes or immunopositive areas of fibrin or GPIIb/IIIa did not differ among the groups (Figure 3D).

### 3.5 Oral administration of FXIa inhibitor does not affect bleeding volume and time in rabbits

Skin bleeding was induced by an incision in both legs of the rabbits after oral administration of the solvent, FXIa inhibitor, and FXa inhibitor. However, the oral administration of ONO-1600586 did not affect the bleeding time or volume. Oral administration of rivaroxaban prolonged bleeding time and increased bleeding volume dose-dependently. Furthermore, a dose of 15 mg/kg rivaroxaban significantly prolonged the bleeding time and increased the bleeding volume compared with the control (Figure 3E).

### 3.6 Oral administration of FXIa and FXa inhibitors suppresses stasis-induced venous thrombus formation in rabbits

**Figure 4:**
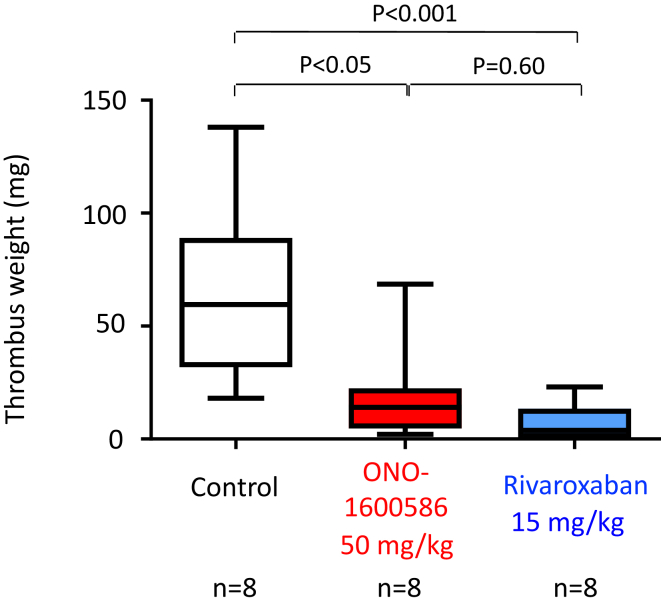
Weight of unfixed stasis-induced venous thrombus in rabbits. Oral administration of ONO-1600586 (50 mg/kg) and rivaroxaban (15 mg/kg) similarly reduced venous thrombus weight compared with that of the control (Kruskal–Wallis test with Dunn’s multiple comparison test; *n* means the number of animals).

Given that DVT is considered to be formed mostly under blood stasis, we additionally examined the roles of FXIa and FXa in stasis-induced thrombus formation in the rabbit jugular vein at 6 hours. We evaluated PT and aPTT before and 1, 3, and 7 hours after the oral administration of the solvent, FXIa inhibitor (ONO-1600586), and FXa inhibitor (rivaroxaban) during venous thrombus formation. ONO-1600586 (50 mg/mL) prolonged aPTT but not PT at 1, 3, and 7 hours, and rivaroxaban (15 mg/mL) prolonged both PT and aPTT at 1, 3, and 7 hours (Supplementary Figure 7). Oral administration of ONO-1600586 (50 mg/kg) and rivaroxaban (15 mg/kg) significantly reduced the venous thrombus weight compared with that of the control (Figure 4). The median value of thrombus weight in ONO-1600586 tended to be higher than that of rivaroxaban, but the values did not reach statistical significance.

### 3.7 Contribution of FXIa and FXa to fibrin formation differs in the *ex vivo* blood flow chamber system

**Figure 5:**
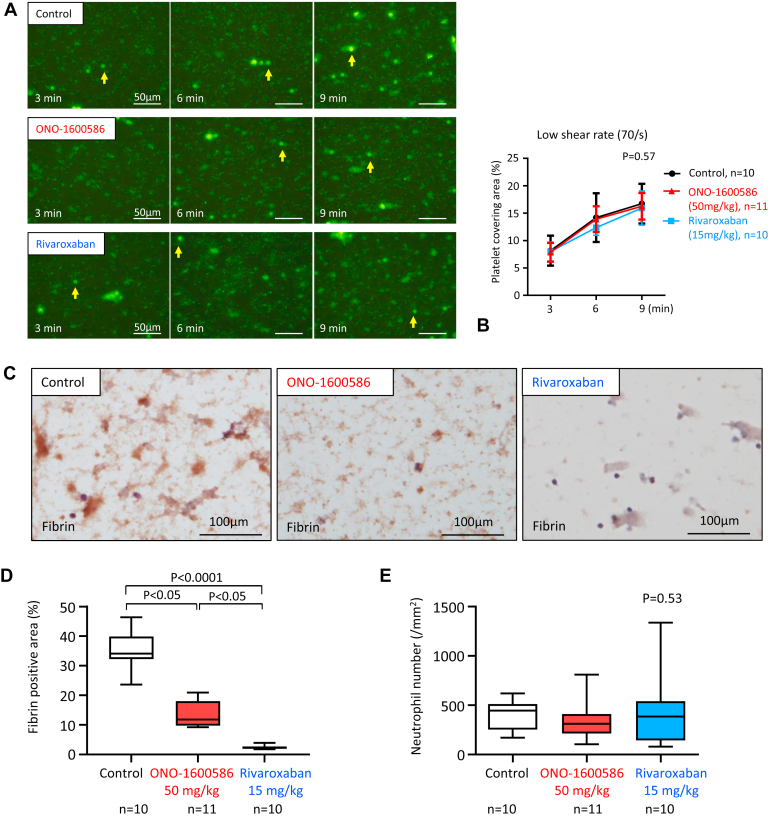
Contributions of activated factor (F)XI and activated FX to *ex vivo* thrombus formation in a flow chamber system. Rabbit blood was collected 75 minutes after oral administration of a solvent (control), ONO-1600586 (50 mg/kg), and rivaroxaban (15 mg/kg). The *ex vivo* blood was perfused in a flow chamber under a low-shear rate of 70/s. (A) Representative fluorescent images under a low-shear rate (70/s). Platelets and leukocytes were labeled with mepacrine, and the fluorescent images were captured 3, 6, and 9 minutes after perfusion. The surface covering area time-dependently increased in all 3 groups. Large fluorescent dots indicate leukocytes (arrows). (B) The surface covering areas under low-shear rate in 3 groups (2-way repeated measure anova with Tukey’s multiple comparison test). (C) Representative immunostaining images of fibrin on the glass coverslips after perfusion. The mesh-like pattern of fibrin formation on islands of aggregated platelets and between the islands in the control. Reduced fibrin formation in ONO-1600586 administration. Absence or little fibrin formation in rivaroxaban administration. (D) Fibrin-immunopositive area on the glass coverslips after *ex vivo* blood perfusion under low-shear rate (Kruskal–Wallis test with Dunn’s multiple comparison test). (E) The number of leukocyte adhesions after *ex vivo* blood perfusion under low-shear rate (Kruskal–Wallis test).

We examined the effect of FXIa and FXa on thrombus formation under low-shear conditions using a flow chamber system. Rabbit blood samples were obtained 75 minutes after the oral administration of the solvent, FXIa inhibitor (ONO-1600586, 50 mg/kg), and FXa inhibitor (rivaroxaban, 15 mg/kg). The oral administration of these doses of inhibitors comparably inhibited venous thrombus formation in rabbits. Additionally, the surface covering areas time-dependently increased in the 3 groups but did not differ among the groups (Figure 5A, B). After perfusion, we stained the thrombus on the coverslips using an antifibrin antibody. Immunostaining showed fibrin formation on the aggregated platelets and between the aggregated sites in the mesh pattern (Figure 5C). Furthermore, the oral administration of ONO-1600586 (50 mg/kg) significantly but incompletely inhibited fibrin formation in the flow chamber, whereas the oral administration of rivaroxaban (15 mg/kg) markedly decreased fibrin formation (Figure 5C, D). Moreover, the oral administration of ONO-1600586 and rivaroxaban did not affect neutrophil attachment on the thrombus (Figure 5E).

### 3.8 Contributions of FXIa and FXa to *in vitro* human thrombus formation in a flow chamber system

**Figure 6:**
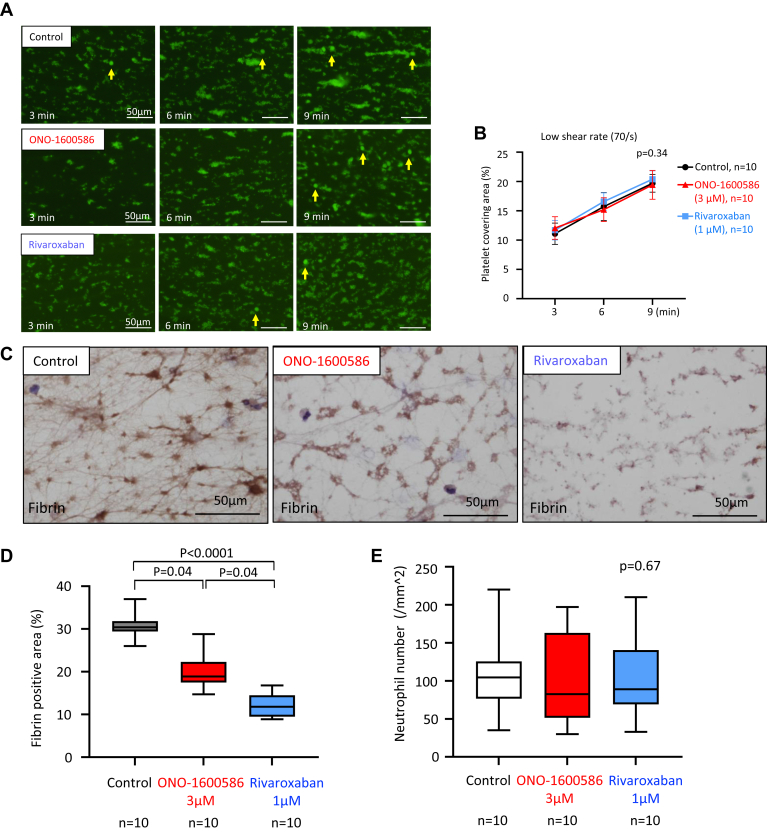

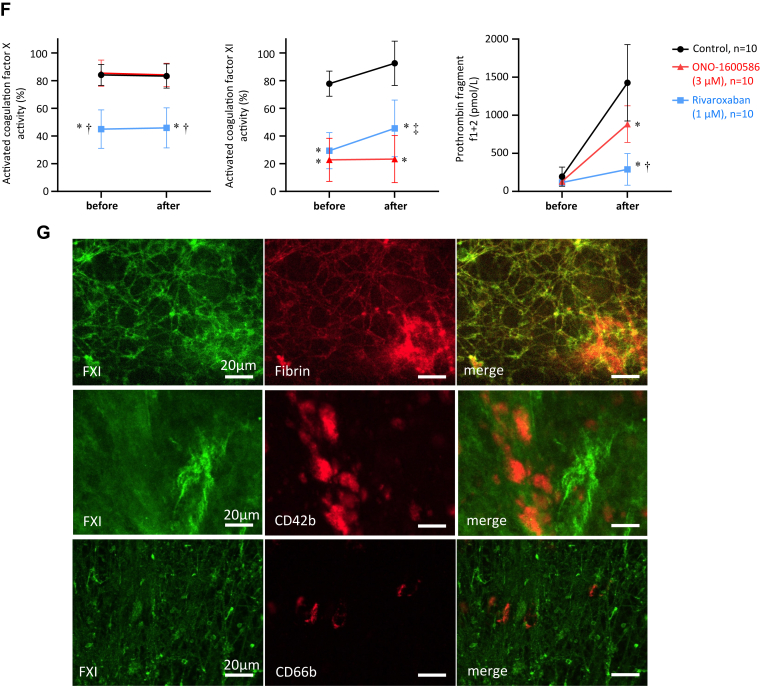
Effects of ONO-1600586 on human activated factor (F)XI (FXIa) and *in vitro* thrombus formation. The human blood was perfused in a flow chamber under a low-shear rate of 70/s. (A) Representative fluorescent images under a low-shear rate (70/s). Platelets and leukocytes were labeled with mepacrine, and the fluorescent images were captured 3, 6, and 9 minutes after perfusion. The surface covering area time-dependently increased in all 3 groups. Large fluorescent dots indicate leukocytes (arrows). (B) The surface covering areas under a low-shear rate in the control (solvent), ONO-1600586, and rivaroxaban addition (2-way repeated measure anova with Tukey’s multiple comparisons test). (C) Representative immunostaining images of fibrin on the glass coverslips after perfusion. ONO-1600586 or rivaroxaban addition decreased fibrin formation. (D) Fibrin-immunopositive area after human blood perfusion under a low-shear rate (Kruskal–Wallis test with Dunn’s multiple comparison test). (E) The number of leukocyte adhesions after human blood perfusion under a low-shear rate (Kruskal–Wallis test). (F) Activated FX activity, FXIa activity, and levels of prothrombin fragment 1 + 2 before and after perfusion of human blood (2-way repeated measure anova with Tukey’s multiple comparison test). ∗*P* < .0001 vs control; †*P* < .0001 vs ONO-1600586; ‡*P* < .01 vs ONO-1600586. (G) Representative immunofluorescent images of the glass coverslips after perfusion. The upper row shows CF488-labeled FXI (green), CF568-labeled fibrin (red), and merged images. The second row shows CF488-labeled FXI, CD42b, and merged images. The lower row shows CF488-labeled FXI, CF568-labeled CD66b, and merged images. FXI was closely localized with fibrin and partly around CD42b, a marker of platelets, but hardly localized with CD66b, a marker of neutrophils.

To examine the effects of FXIa and FXa on human thrombus formation *in vitro*, we added a control solvent, FXIa inhibitor (ONO-1600586, 3 μmol/L), or FXa inhibitor (rivaroxaban, 1 μmol/L) to human blood. We measured platelet thrombus formation during human blood perfusion, as well as fibrin formation and leukocyte adhesion after the perfusion, and assessed FX and FXI activities and thrombin generation before and after the perfusion in a flow chamber system. The dose of ONO-1600586 prolonged aPTT (78 ± 2 seconds, *n* = 8, 2.2-fold compared with control) but did not affect PT (10 ± 0.6 seconds, *n* = 8, 1.0-fold compared with control), as well as oral administration of ONO-1600586 (50 mg/kg) in rabbits. The dose of rivaroxaban prolonged PT (19 ± 3 seconds, *n* = 8, 1.9-fold compared with control) and aPTT (62 ± 5 seconds, *n* = 8, 1.8-fold compared with control), as well as oral administration of rivaroxaban (15 mg/kg) in rabbits. The inhibition of FXIa and FXa did not affect the platelet covering area under low-shear conditions (Figure 6A, B). ONO-1600586 (3 μM) significantly but incompletely inhibited fibrin formation in the flow chamber, whereas rivaroxaban (1 μmol/L) markedly decreased fibrin formation under low-shear conditions (Figure 6C, D). The inhibition of FXIa and FXa did not affect leukocyte adhesion under low-shear conditions (Figure 6E). FXIa activity, rather than FXa activity, in the plasma after perfusion was higher than that in the plasma before perfusion. FXIa inhibition suppressed the enhanced FXIa activity. FXa inhibition reduced FXa activity and plasma levels of prothrombin fragment 1 + 2 after perfusion, while FXIa inhibition significantly reduced plasma levels of prothrombin fragment 1 + 2, but not as much as FXa inhibition (Figure 6F).

To identify FXI localization in the *in vitro* human thrombus, we performed double immunofluorescence for FXI-fibrin, FXI-CD42b (platelet), or FXI-CD66b (neutrophil). FXI was mainly colocalized with fibrin and partly around the aggregated platelets (CD42b) but not with neutrophils (CD66b) in the *in vitro* thrombus (Figure 6G).

## 4 Discussion

This study showed that FXI was mainly localized in the nonorganizing area of human DVT and was closely distributed in fibrin using immunohistochemistry, immunofluorescence, and *in situ* nanogold labeling methods. Additionally, an orally administered direct FXIa inhibitor, ONO-1600586, rather than the FXa inhibitor, did not enhance bleeding under suppressed venous thrombus formation in rabbits, whereas ONO-1600586 incompletely inhibited fibrin formation *ex vivo* under low-shear conditions. FXIa inhibition suppressed human FXIa activity and reduced thrombin generation and fibrin formation during human blood perfusion, but not as much as FXa inhibition.

FXI was present in all aspirated DVT, mainly localized in fibrin- and erythrocyte-rich nonorganizing areas and closely distributed in fibrin. The presence of intrinsic coagulation factors in human DVT has been reported previously. Sugita et al. [20] examined the localization of FVIII, von Willebrand factor, and fibrin in DVT and found colocalization of FVIII with platelets, von Willebrand factor, and fibrin. The results suggest that FXI localization in DVT is not always similar to that of FVIII. FXI circulates in the plasma, is stored in platelets, and is secreted upon platelet activation [21]. Although FXIa activity and antigen have been described in washed platelet suspensions, FXIa activity constitutes approximately 0.5% of the FXIa activity in normal plasma [21]. Additionally, plasma FXI levels are associated with DVT incidence [[5], [6], [7], [8], [9]]. FXII is considered to contribute to venous thrombus growth through the activation of DNA in neutrophil extracellular traps (NETs) [22]. Predominant colocalization with fibrin and focal colocalization with DNA and FXII are comparable to the results of this study, and FXI was abundant in the nonorganizing areas compared with the organizing area in this study. FXI was also colocalized with fibrin and partly around the aggregated platelets in *in vitro* thrombus formation under low-shear conditions. Our findings suggest that FXI participates in human DVT formation and may be degraded during the organizing process, similar to other thrombus components, such as erythrocytes, fibrin, platelets, and neutrophils.

There were a few areas of colocalization of FXI and neutrophils in human DVT and *in vitro* thrombi. Shi et al. [23] examined the role of neutrophils and NETs in clot formation, and the procoagulant effects of partially activated neutrophils and neutrophil supernatant were mediated via FXI in clotting kinetics. The results suggest that any proteins released from neutrophils can interact with FXI in the fluid phase but not on the neutrophil surface. Shi et al. [23] also showed that the procoagulant effects of NETs were independent of FXI, FXII, or FVII. FXI and FXII deficiency did not affect stasis-induced venous thrombus formation in mice in the presence of NETs [24]. These studies suggest that NETs contribute to stasis-induced thrombus formation independent of FXI and FXII.

The direct oral FXIa inhibitor ONO-1600586 suppressed endothelial denudation- or stasis-induced venous thrombus formation without excessive bleeding in rabbits. Notably, these findings are comparable to those in FXI-deficient mice [10], anti-FXI antibodies [13,25,26], FXI antisense oligonucleotides [12,27], and small-molecule FXIa inhibitors [11]. However, ferric chloride [10,12] and silk thread [11] are not physiological initiators of venous thrombus formation; therefore, the data should be interpreted cautiously. Our study strongly supports the hypothesis that the enzymatic activity of FXIa contributes to venous thrombus formation *in vivo*. The relative areas of erythrocytes, fibrin, and platelets in the venous thrombi did not differ between groups, suggesting that their compositions are independent of the venous thrombus size *in vivo*. A previous study showed that an anti-FXI antibody reduced both platelet and fibrin content in venous thrombi compared with controls [13]. The difference might be because of FXIa inhibition rather than FXI inhibition.

Orally available small-molecule inhibitors are more suitable for the long-term prevention of venous thrombosis than large-molecule inhibitors. I.v. administration of BMS-262084, which is an irreversible small-molecule FXIa inhibitor, reduced silk thread-induced thrombus in the inferior vena cava of rabbits [11]. Therefore, this study is the first to show that the oral administration of a FXIa inhibitor is effective for venous thrombus formation *in vivo*. Although ONO-1600586 can inhibit plasma kallikrein, the higher IC_50_ value of human kallikrein compared with that of human FXIa suggests that the antithrombotic effect mostly reflects those of FXIa inhibition in rabbits.

Clinical and experimental studies have shown that FXI deficiency and inhibition have a lower bleeding tendency than FX deficiency and inhibition [8,9,28]. FXIa and FXa inhibition showed different hemostatic effects with similarly reduced venous thrombus formation in this study. These results are consistent with those of a previous study [26]. Antihuman FXI antibodies mildly inhibited ferric chloride-induced venous thrombus formation without altering the tail cut bleeding time in mice, whereas enoxaparin strongly inhibited venous thrombus formation and prolonged bleeding time [26]. However, why FXI/FXIa inhibition does not result in excess bleeding remains unclear. Our *ex vivo* and *in vitro* studies showed that FXIa inhibition significantly but incompletely inhibited mural fibrin formation under low-shear conditions compared with FXa inhibition (Figure 5C, D and Figure 6C, D). Therefore, the limited contribution of fibrin formation on the vascular surface may explain the differences in bleeding tendencies between FXI and FX deficiency and inhibition.

Oral and parenteral FXI/FXa inhibitors have been evaluated in phase I and II clinical trials and are currently undergoing phase III evaluation. The present FXIa inhibitor ONO-1600586 suppressed human FXIa activity, thrombin generation, and fibrin formation under low-shear conditions (Figure 6A). These results suggest that human FXI can be activated via thrombin during venous thrombus formation and that the small-molecular inhibitor ONO-1600586 can inhibit FXIa activity under low-shear conditions. The phase I study on an ONO-1600586–related product, ONO-7684, which is an oral FXIa inhibitor, showed safety and tolerability for approximately 14 days in the fed state in healthy individuals [29]. An open-labeled noninferiority phase II trial compared osocimab, human monoclonal anti-FXI antibody, enoxaparin, and apixaban for thromboprophylaxis in patients who had undergone knee arthroplasty [30]. A single infusion of osocimab showed noninferiority compared with enoxaparin for the incidence of VTE at 10 to 13 days. The major or clinically relevant nonmajor bleeding rate was approximately 4.7%, 5.9%, and 2% in patients receiving osocimab, enoxaparin, and apixaban, respectively. The Safety of the Oral Factor XIa Inhibitor Asundexian Compared With Apixaban in Patients With Atrial Fibrillation trial compared the efficacy and safety of asundexian, which is a small-molecule FXIa inhibitor, and apixaban. Although underpowered for efficacy, asundexian (50 mg, once daily) showed a lower bleeding rate (0.4%) than apixaban (2.4%) [31]. Therefore, further studies are needed to establish the efficacy and safety of FXI/FXIa inhibitors in humans.

This study had some limitations. First, the sample size of human-aspirated DVT was small. However, the consistent presence of FXI in fibrin-rich areas suggests that FXI plays a role in fibrin formation during DVT. CD34 is not a specific maker of the endothelium. Because platelets express CD31, an endothelial marker, we used an anti-CD34 antibody for endothelialization. Second, rabbit FXI lacks Cys321 and, therefore, is circulating as a noncovalently associated dimer [32]. This may affect the functional difference between rabbit and human FXIa. However, ONO-1600586 similarly prolonged aPTT in humans and rabbits. Third, we only semiquantified the immunohistochemical and immunofluorescent colocalized areas; hence, the lack of exact values is shown in Figure 1E. Fourth, *in vitro* experiments may not accurately reflect thrombus formation *in vivo* because we perfused anticoagulated rabbit and human blood. The anticoagulant will lead to delayed thrombus formation and subsequent fibrin generation; therefore, the results showing reduced or abolished fibrin formation with the FXIa and FXa inhibitors may be overestimated. Fifth, the thrombogenicity of blood may differ between humans and rabbits. We required different pretreatments and anticoagulation in the flow chamber experiments with human and rabbit blood.

## 5 Conclusions

FXI localization may provide a rationale for FXI/FXIa inhibition to prevent human DVT, and preserved fibrin formation under FXIa inhibition may be associated with a minor hemostatic role of FXIa.
