# Supplementary material for: Factor XI localization in human deep venous thrombus and function of activated factor XI on venous thrombus formation and hemostasis
Source: Res Pract Thromb Haemost. 2025 Mar 3;9(2):102720. doi: 10.1016/j.rpth.2025.102720 (PMC11999338; doi:10.1016/j.rpth.2025.102720)
Supplement: Supplementary Figure 1 [file mmc1.pdf]

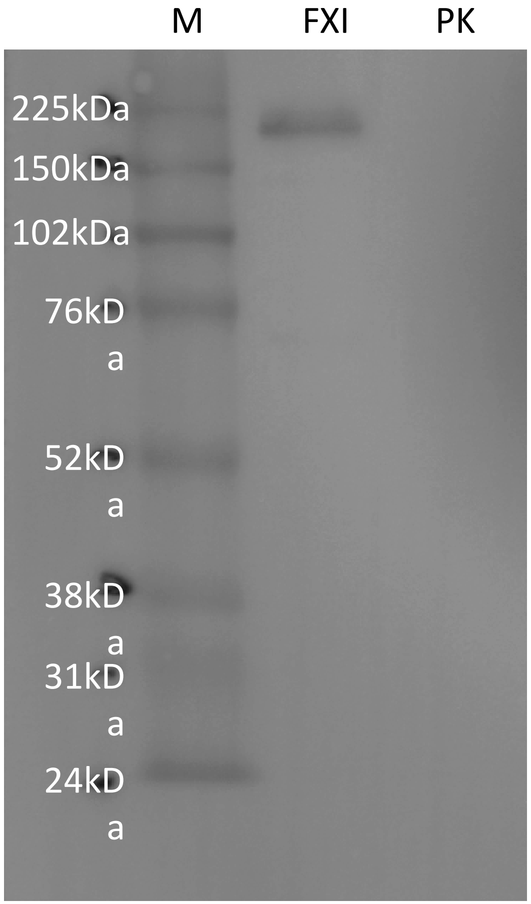

Supplementary Figure 1. Western blot using anti-FXI antibody.
M: ECL Rainbow Marker-Full range (Cytiva, Tokyo, Japan)
FXI: purified human FXI (HFXI 1111, Enzyme Research Laboratories Ltd., Swansea, UK) ;
PK: purified human prekallikrein (HPK 1302, Enzyme Research Laboratories Ltd.)
The western blotting using anti-sheep FXI antibody (sheep polyclonal, LS-B10243; LifeSpan, Inc. Seattle, USA, final concentration 10µg/mL) was performed under non-reducing condition.
