# Supplementary material for: Factor XI localization in human deep venous thrombus and function of activated factor XI on venous thrombus formation and hemostasis
Source: Res Pract Thromb Haemost. 2025 Mar 3;9(2):102720. doi: 10.1016/j.rpth.2025.102720 (PMC11999338; doi:10.1016/j.rpth.2025.102720)
Supplement: Supplementary Figure 1 [file mmc2.pdf]

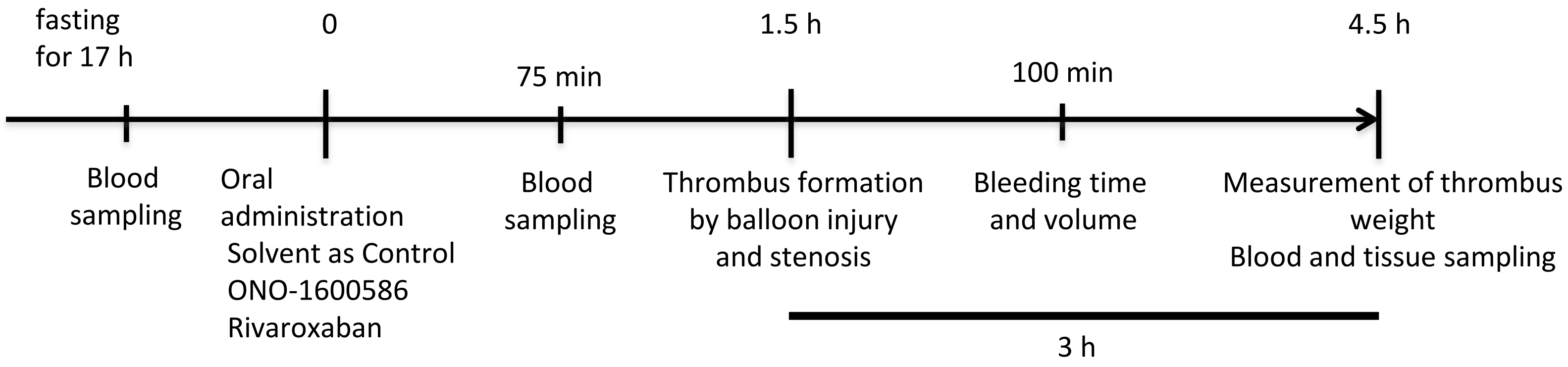

Supplementary Figure 2. A protocol of rabbit model of jugular vein thrombosis with endothelial denudation and luminal stenosis, and skin bleeding.

ONO-1600586, an activated factor XI inhibitor
