# Supplementary material for: Factor XI localization in human deep venous thrombus and function of activated factor XI on venous thrombus formation and hemostasis
Source: Res Pract Thromb Haemost. 2025 Mar 3;9(2):102720. doi: 10.1016/j.rpth.2025.102720 (PMC11999338; doi:10.1016/j.rpth.2025.102720)
Supplement: Supplementary Figure 1 [file mmc3.pdf]

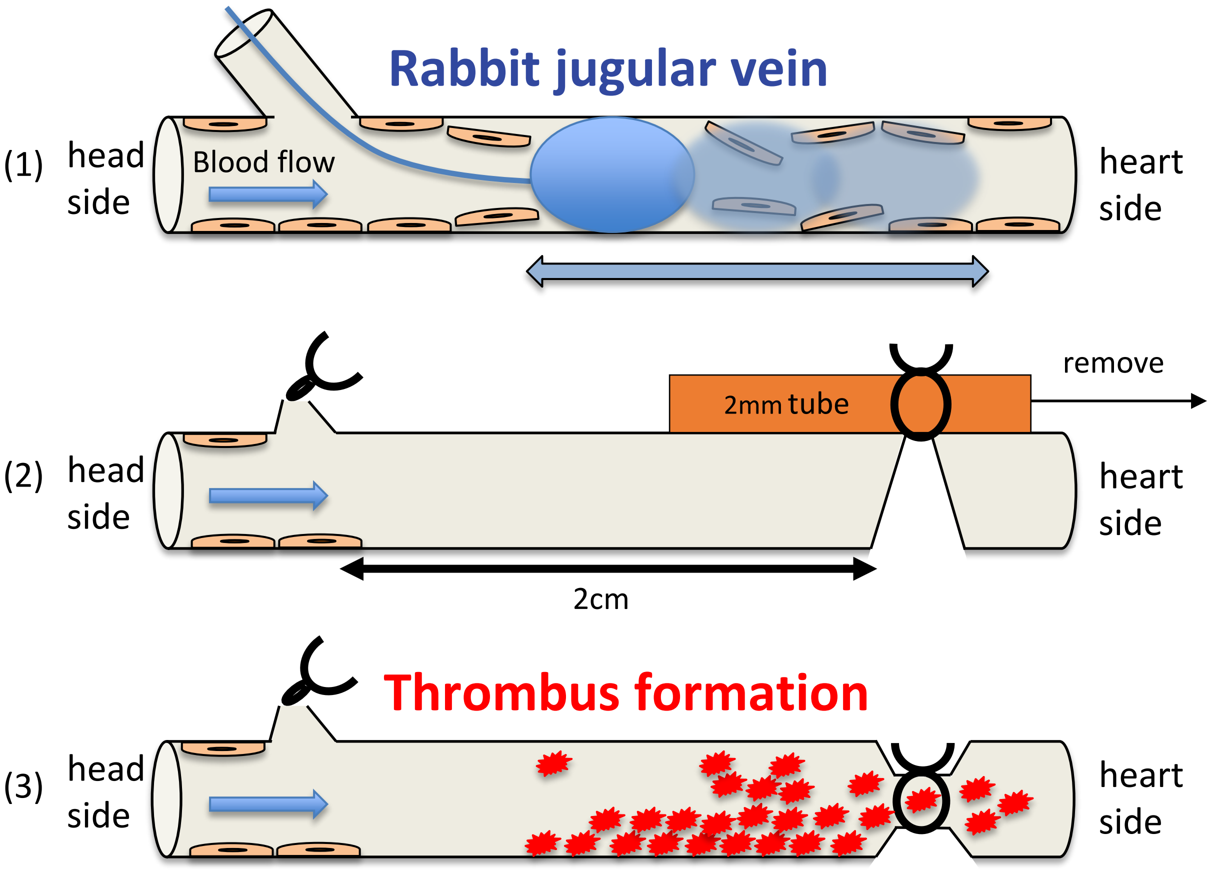

Supplementary Figure 3. Procedure of rabbit jugular vein thrombus model with endothelial denudation and luminal and stenosis.

(1) Balloon catheter (3F) inserted 5 cm into the jugular vein via the distal branch was inflated three times (air volume, 0.4 mL) for endothelial denudation.
(2) To induce luminal stenosis at the jugular vein 2 cm-proximal from the branch, both jugular vein and a polyethylene tube of 2 mm in outer diameter were ligated, and the tube was removed.
(3) Thrombi were sampled and weighed 3 h after thrombus formation (4.5 h after administration of solvent or inhibitors).
