# Supplementary material for: Factor XI localization in human deep venous thrombus and function of activated factor XI on venous thrombus formation and hemostasis
Source: Res Pract Thromb Haemost. 2025 Mar 3;9(2):102720. doi: 10.1016/j.rpth.2025.102720 (PMC11999338; doi:10.1016/j.rpth.2025.102720)
Supplement: Supplementary Figure 1 [file mmc5.pdf]

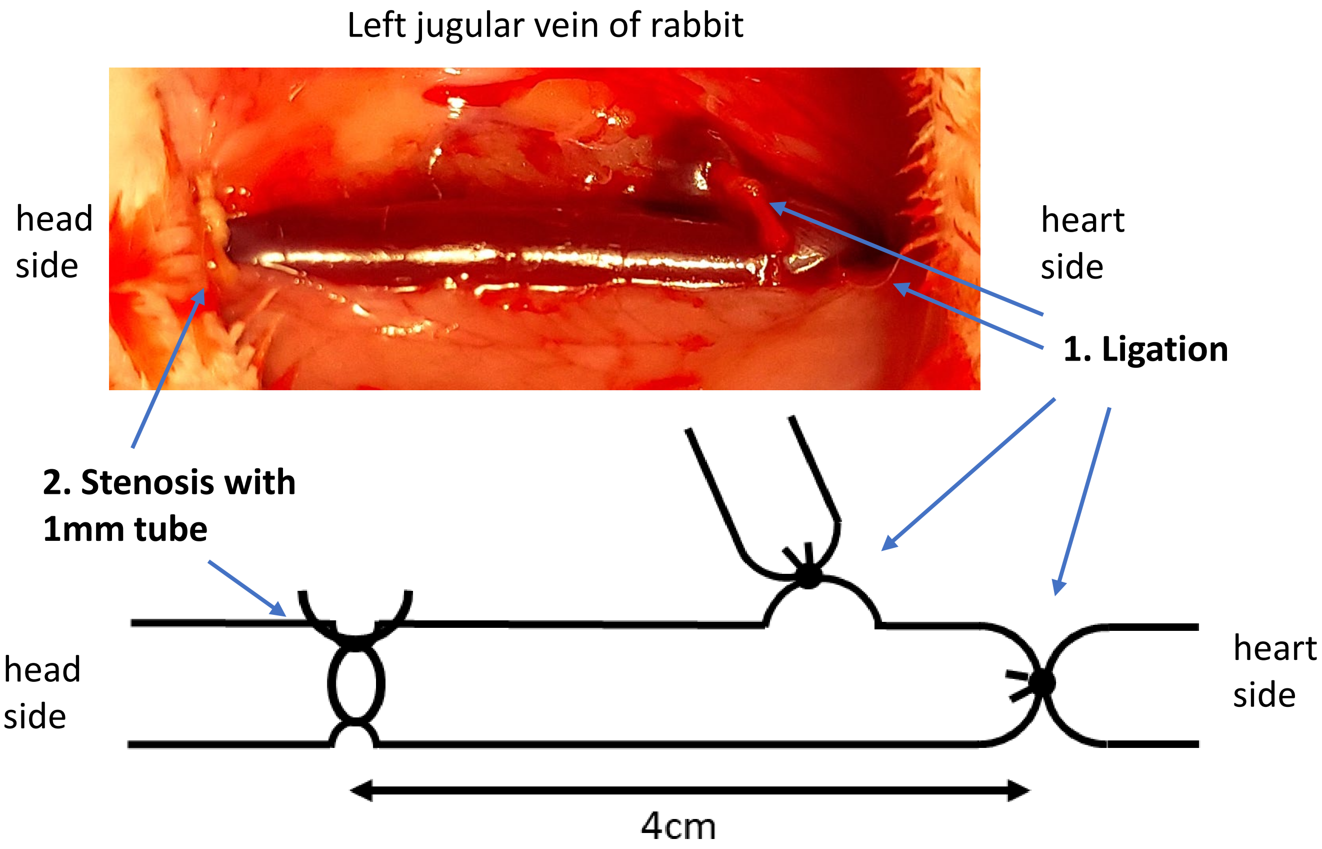

Supplementary Figure 5. A procedure of stasis-induced thrombus formation model in rabbit jugular vein.

We exposed both jugular veins of the rabbit and ligated the branches and heart side of the jugular vein. The head side of the jugular vein with a polyethylene tube of 1 mm in the outer diameter was ligated, and the tube was removed.
