# Supplementary material for: Factor XI localization in human deep venous thrombus and function of activated factor XI on venous thrombus formation and hemostasis
Source: Res Pract Thromb Haemost. 2025 Mar 3;9(2):102720. doi: 10.1016/j.rpth.2025.102720 (PMC11999338; doi:10.1016/j.rpth.2025.102720)
Supplement: Supplementary Figure 1 [file mmc6.pdf]

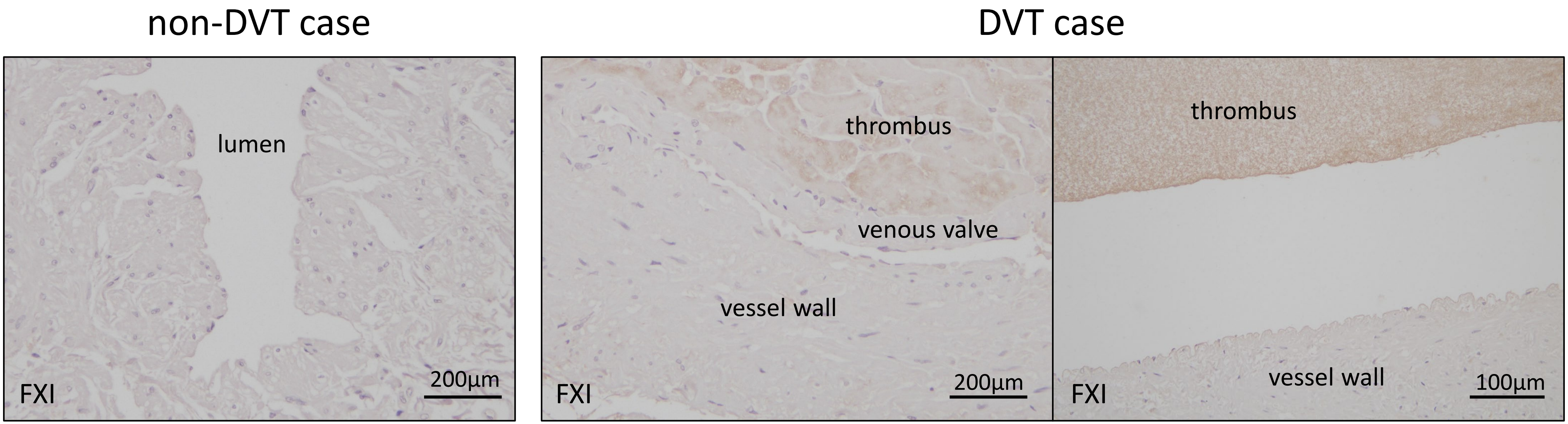

Supplementary Figure 6. Representative images of immunohistochemistry of factor XI (FXI) in human vein with or without deep vein thrombosis (DVT).

Deep vein thrombi but not venous wall are immunopositive for FXI.
