# Supplementary material for: Factor XI localization in human deep venous thrombus and function of activated factor XI on venous thrombus formation and hemostasis
Source: Res Pract Thromb Haemost. 2025 Mar 3;9(2):102720. doi: 10.1016/j.rpth.2025.102720 (PMC11999338; doi:10.1016/j.rpth.2025.102720)
Supplement: Supplementary Figure 1 [file mmc7.pdf]

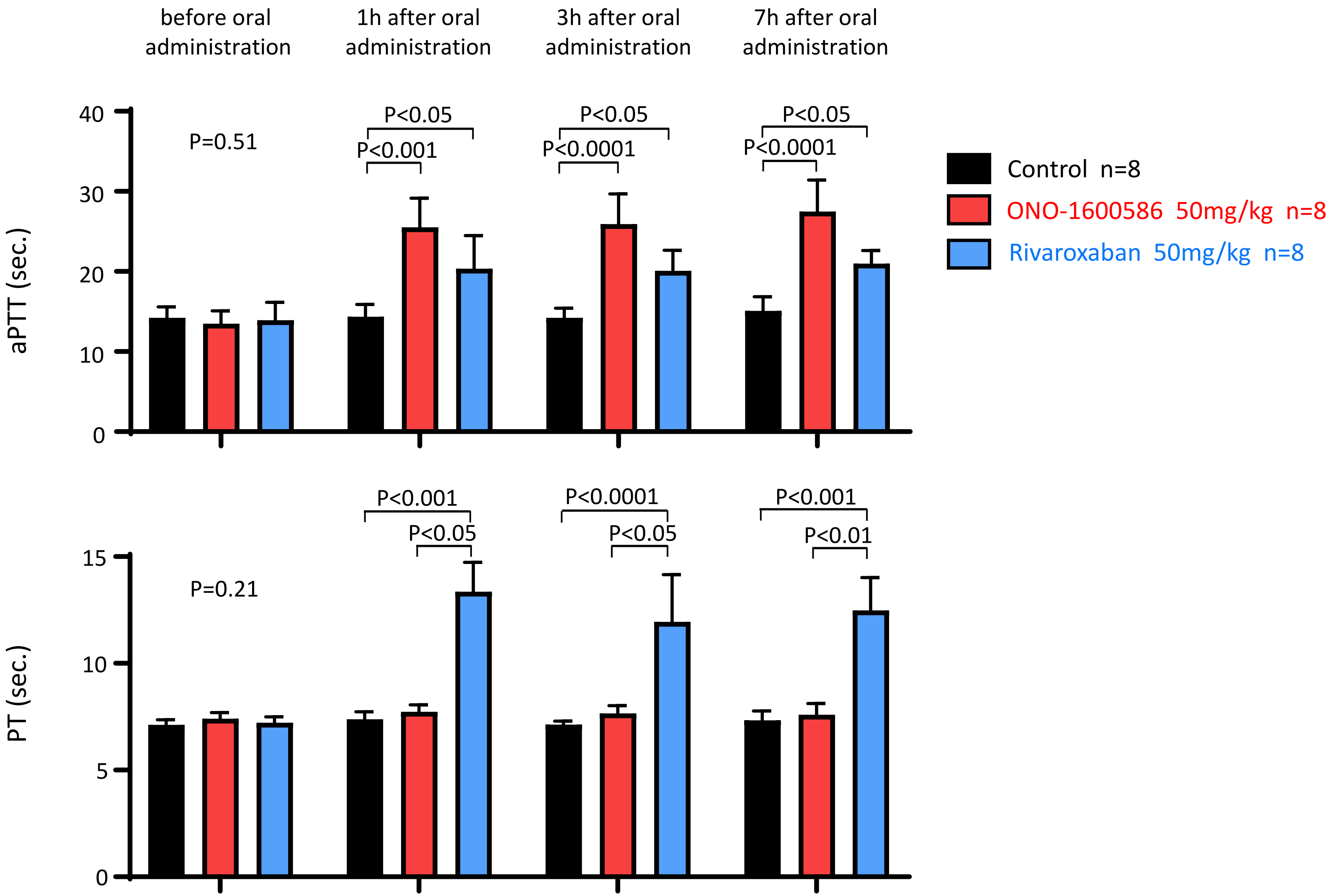

Supplementary Figure 7. prothrombin time (PT) and activated partial thromboplastin time (aPTT) in rabbit model of stasis-induced venous thrombus formation.

PT and aPTT before, 1 h (before thrombus formation), 3h (2h after thrombus formation) and 7 h (6 h after thrombus formation) after oral administration of solvent (control) and activated factor XI (FXIa) (ONO-1600586, 50 mg/kg) and factor X (FX) inhibitors (Rivaroxaban, 15 mg/kg). Kruskal–Wallis test with Dunn's multiple comparison test. N means number of animals.
