# Supplementary material for: Factor XI localization in human deep venous thrombus and function of activated factor XI on venous thrombus formation and hemostasis
Source: Res Pract Thromb Haemost. 2025 Mar 3;9(2):102720. doi: 10.1016/j.rpth.2025.102720 (PMC11999338; doi:10.1016/j.rpth.2025.102720)
Supplement: Supplementary Table 1 [file mmc8.pdf]

**Supplementary Table 1. Clinical background of DVT patients (n=15)**

| | | |
|---|---|---|
| Age, median (range; years) | 54 | (20-78) |
| Male sex, n (%) | 8 | (53%) |
| Obesity; BMI >25, n (%) | 3 | (20%) |
| Smoking, n (%) | 8 | (53%) |
| Complication | | |
| Posttraumatic, n (%) | 4 | (27%) |
| Cancer, n (%) | 2 | (13%) |
| Antiphospholipid syndrome, n (%) | 0 | (0%) |
| Medication | | |
| Aspirin, n (%) | 2 | (13%) |
| Warfarin, n (%) | 3 | (20%) |
| Chemotherapy, n (%) | 0 | (0%) |
| Steroid, n (%) | 3 | (20%) |
| Treatment period of heparin infusion before aspiration, median (range; days) | 4 | (0-30) |
| Laboratory data before thrombus aspiration, median (range) | | |
| Complete blood cell count | | |
| White blood cell (x$10^3$/µL) | 6.8 | (4.1-14.4) |
| Red blood cell (x$10^6$/µL) | 3.68 | (2.3-4.88) |
| Hemoglobin (g/dL) | 11.8 | (7.5-17.1) |
| Platelet (x$10^3$/µL) | 225 | (117-724) |
| Coagulation | | |
| D-dimer (µg/mL) | 13 | (4.5-76.2) |
| PT-INR (INR) | 1.11 | (0.9-2.1) |
| aPTT (sec.) | 31.7 | (23.5-45.2) |
| Activated protein S (%) | 78 | (28-144) |
| Activated protein C (%) | 96 | (29-134) |
| Antithrombin III activity (%, n=14) | 91 | (35-126) |

aPTT, activated partial thromboplastin time; BMI, body mass index; DVT, deep vein thrombosis;
PT-INR, prothrombin time-international normalized ratio;
