# Supplementary material for: Factor XI localization in human deep venous thrombus and function of activated factor XI on venous thrombus formation and hemostasis
Source: Res Pract Thromb Haemost. 2025 Mar 3;9(2):102720. doi: 10.1016/j.rpth.2025.102720 (PMC11999338; doi:10.1016/j.rpth.2025.102720)
Supplement: Supplementary Table 1 [file mmc9.pdf]

Supplementary Table 2. Effects of ONO-1600586 on human coagulation enzymes

Human enzymes	IC50 (μM)
Activated factor XI	0.0020
Plasma Kallikrein	0.12
Thrombin	26
Activated factor VII	>33
Activated factor IX	>100
Activated factor XII	>33
Plasmin	67
Urokinase	>33
Tissue plasminogen activator	>33
